# Ultrasensitive sandwich-type electrochemical immunosensor based on trimetallic nanocomposite signal amplification strategy for the ultrasensitive detection of CEA

**DOI:** 10.1038/srep30849

**Published:** 2016-08-04

**Authors:** Lihui Tian, Li Liu, Yueyuan Li, Qin Wei, Wei Cao

**Affiliations:** 1 Key Laboratory of Chemical Sensing & Analysis in Universities of Shandong, School of Chemistry and Chemical Engineering, University of Jinan, Jinan 250022, P. R. China

## Abstract

A novel and ultrasensitive sandwich-type electrochemical immunosensor was designed for the quantitative detection of carcino-embryonic antigen (CEA). This immunosensor was developed by using the trimetallic NiAuPt nanoparticles on graphene nanosheets (NGs) nanosheets (NiAuPt-NGs) as excellent labels and β-cyclodextrin functionalized reduced graphene oxide nanosheets (CD-NGs) as the platform. The CD-NGs with high specific surface area good biocompatibility and the ideal dispersibility was used to capture the primary antibodies (Ab_1_) efficiently. The trimetallic NiAuPt-NGs nanocomposites were used as the labels for signal amplification, showing better electrocatalytic activity towards the reduction of hydrogen peroxide (H_2_O_2_), which is much better than that the monometallic Pt-NGs, bimetallic NiPt-NGs and AuPt-NGs due to the synergetic effect presented in NiAuPt-NGs. The NiAuPt-NGs nanocomposites consist of tightly coupled nanostructures of Au, Ni and Pt, which have neither an alloy nor a core-shell structure. Under the optimal conditions, a linear range from 0.001–100 ng/mL and a low detection limit of 0.27 pg/mL were obtained for CEA. The proposed electrochemical sandwich-type immunosensor may have a promising application in bioassay and it enriches the electrochemical immunoassays.

Tumor markers refer to the specific substances that exist in tumor cells themselves or are secreted by tumor cells^1^. They can reflect the existence and growth of the tumor. Carcino-embryonic antigen (CEA), a widely used tumor maker which is produced in excess in essentially all human colon carcinomas and in a high proportion of carcinomas at many other sites, is widely used for clinical research and early diagnosis according the quantitative in the serum^2^. It is necessary to provide a simple, reliable and sensitive detection method for CEA in clinical research^3^.

Due to the high sensitivity, inherent simplicity, rapid detection, miniaturization, low cost and other advantages, the electrochemical immunosensors have caused great concern and have been developing rapidly in recent years^4^,^5^,^6^. And the electrochemical immunosensors have been developed in many areas not only be applied to biochemistry, clinical diagnosis but also the environmental monitoring food safety and other fields^7^,^8^,^9^,^10^. In order to enhance the sensitivity and selectivity of the biosensors, many methods have been applied to enlarge the signal of the immunosensor especially the nanomaterials with excellent catalytic performance^11^.

In this work, a sandwich-type electrochemical immunosensor based on a novel signal amplification strategy was prepared by using β-cyclodextrin (β-CD) functionalized reduced graphene oxide nanosheets (CD-NGs) as a platform for the immobilization of primary antibobies for the detection of CEA. Graphene nanosheets (NGs), a new two-dimensional (2D) monolayer, have the advantages of large surface area and excellent mechanical properties and high electrical conductivity^12^,^13^,^14^. But during the process of graphene oxide (GO) was reduced to NGs, due to the loss of oxygen containing functional groups, NGs tends to form irreversible agglomerates via van der Waals interactions^15^, and it is difficult for protein molecules to be immobilized on the surface of NGs directly. To overcome this problem, the β-CD was introduced into graphene oxide before the graphene oxide was fully reduced. The introduced β-CD onto the surface of NGs prevented the agglomeration of the NGs improved solubility of the CD-NGs in water. The β-CD which has a hydrophobic inner cavity and a hydrophilic is a kind of toroidal biological macromolecules^16^. The high supramolecular recognition characteristic will enable them to bind selectively with guest molecules to form a stable host-guest complex^17^,^18^. Therefore, β-CD can not only prevent the stacking of NGs but also improve the molecular recognition ability of NGs. In this work, the primary anti-CEA (Ab_1_) which containing amino functional group can enter into the cavities of β-CD to form stable host-guest inclusion complexes by the host-guest interaction^19^. The CD-NGs with a good electron transfer ability, biocompatibility and large specific surface area, is used as a platform for the immobilization of Ab_1_, which enhances the loading of antibodies improving the sensitivity of the electrochemical immunosensor.

The noble metals (Au, Pd, Pt etc.) doped with non-noble metals (Fe, Co, Ni etc.) is an excellent approach to enhance the catalytic activity and the sensitivity of nanomaterials though their synergistic effect^20^,^21^. In recent years, it has been extensively used in bioaffinity sensors due to their superior electrocatalytic activity for the reduction of H_2_O_2_^22^,^23^. In order to designed more effective immunosensors, we used NiAuPt-NGs which show excellent electrocatalytic activity for the reduction of H_2_O_2_ as labels of secondary anti-CEA (Ab_2_), and the immunosensor could build a direct relationship between concentrations of antigens and electrochemical signal intensity^24^. According the reports of the documents before, the monometallic, bimetallic and trimetallic nanomaterial have been used to design for the immunosensors^25^,^26^,^27^,^28^, but the special trimetallic NiAuPt-NGs nanocomposites have not been used to design for the immunosensors. The NiAuPt nanoparticles grow on the NGs after the chemical reduction of their precursors. They consist of tightly coupled nanostructures of Ni, Au and Pt, which have neither an alloy nor a core-shell structure. Trimetallic NiAuPt nanoparticles possess better resistance against electromigration than bimetallic or monometallic nanoparticles. And the NiAuPt-NGs displayed an excellent electrocatalytic performance to H_2_O_2_, which the electrochemical signal for H_2_O_2_ is more than 7 times higher than that on the monometallic Pt-NGs, 3 times higher than that on the bimetallic NiPt-NGs, and almost 2 times higher than that on the bimetallic AuPt-NGs. The high electrocatalytic activity of NiAuPt-NGs is attributed to the synergetic effect of the three nanostructured metals. Thereby, the electrocatalysis sensitivity of the immunosensor is obviously increased. Herein, an innovative sandwich-type electrochemical immunosensor was fabricated for the sensitive detection of CEA based on the trimetallic NiAuPt-NGs nanocomposite. Figure 1 shows the illustration of the fabrication procedure of the sandwich-type immunosensor.

The immunosensor is fabricated by the following processes: (1) The CD-NGs with great specific surface area and high electrical conductivity is easy to capture Ab_1_ owing to the host-guest interaction. And antigens were captured onto the antibody sites by a specific antigen-antibody bond. (2) NiAuPt-NGs as the signal tag could combine Ab_2_ directly forming Ab_2_@NiAuPt-NGs which could be successively conjugated with the antigen via immunoreaction. The proposed immunosensor showed a high current response, a relatively wide linear range and a low detection limit for the detection of CEA.

## Results and Discussion

### Characterization of the CD-NGs

**Figure 2:**
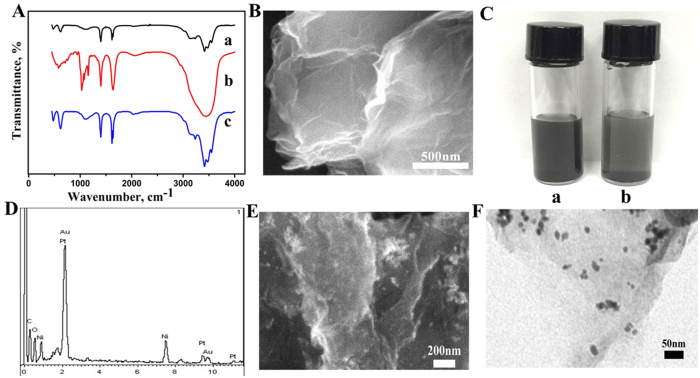
(**A**) FT-IR spectra of NGs (a), β-CD (b), CD-NGs (c). (**B**) The SEM image of CD-NGs. (**C**) The dispersibility of the CD-NGs (a) and NGs (b) in water. (**D**) EDX spectrum of NiAuPt-NGs. (**E**) The SEM image of spectrum of NiAuPt-NGs. (**F**) TEM image of NiAuPt-NGs.

In this work, NGs was well modified with β-CD, to prove this, FT-IR spectrum of NGs (curve a), β-CD (curve b) and CD-NGs (curve c) was recorded as shown in Fig. 2A. In the spectrum of pure NGs the characteristic absorption bands at 3413 cm^−1^ (O-H stretching vibration), 1109 cm^−1^ (the bending vibrations of coupled C-O-C stretching), 1630 cm^−1^ (the bending vibrations of coupled O-H) can be found. And from the FT-IR spectrum of CD-NGs the main absorption peaks of pure NGs was also appeared. Moreover CD-NGS exhibits absorption peaks at 2000 cm^−1^ (C = C bending vibrations), 943 cm^−1^ (skeletal vibration), 709 cm^−1^ (pyranose ring vibration), 579 cm^−1^ (pyranose ring vibration) which is found to be conformity with spectroscopic characterizations of β-CD. It can be seen that β-CD molecules are attached to the surface of NGs. For further identifying that the β-CD molecules have combined with NGs, CD-NGs was characterized using UV-vis spectroscopy as shown in Supplementary Fig. S1D. According to the UV-vis spectroscopy analysis, the β-CD solution had a major peak at 266 nm (curve b). No absorption peak at 266 nm was observed for the NGs (curve c). And an absorption peak at 266 nm was also observed for the CD-NGs (curve a), indicating the successful synthesis of CD-NGs. The Fig. 2B shows the SEM image of the CD-NGs. It found that the disorder and folded multilayer surface of the CD-NGs, which like layers of transparent tulle. The TEM of the NGs, CD-NGs and the SEM of the NGs were obtained and the result was shown in Supplementary Fig. S1. It can be seen that from the Fig. S1 there is no significant difference in structure between CD-NGs and NGs. As the β-CD was well dispersed among the whole NGs plane, the thickness of CD-NGs will be influenced but not too much. The GO with lots of oxygen-containing groups has favorable dispersibility in water. Compared with it, the dispersion of the NGs which prepared by chemical reduction of GO is poor because of the absence of the oxygenic groups. But after NGs was modified with β-CD, it remains soluble in water and does not aggregate for a long time. The 2 mg of NGs and CD-NGs were added into 2 mL of ultrapure water respectively in the glass vial then ultrasonicated for 10 minutes. After half an hour we took a picture for the two vials. The Fig. 2C shows the dispersibility of the CD-NGs (a) and NGs (b) in water. From the picture we could find that the color of the bottle (a) is darker than bottle (b), proving that CD-NGs has better dispersibility than pure NGs.

### Characterization of the NiAuPt-NGs nanocomposite

The microstructure of the NiAuPt-NGs nanocomposites were investigated by SEM and TEM. Figure 2E is the SEM image of NiAuPt-NGs nanocomposites. These bright spots are the trimetallic nanoparticles in the SEM image, and the size of the NiAuPt nanoparticles is about 20 nanometers. The SEM image of NiAuPt-NGs nanocomposites indicate that the nanoparticles are well dispersed on the NGs. Figure 2F shows the TEM image of the NiAuPt-NGs nanocomposites, and those black dots are the trimetallic NiAuPt nanoparticles. From the picture NiAuPt nanoparticles can be seen clearly and they are uniformly distributed on the surface of NGs. Figure 2D shows the EDX spectrum of the prepared NiAuPt-NGs nanocomposites. The presence of the Ni, Pt, Au element in the EDX spectrum is proof that the preparation of the composite material was very successful.

### The electrochemical characterization of NiAuPt-NGs nanocomposites

**Figure 3:**
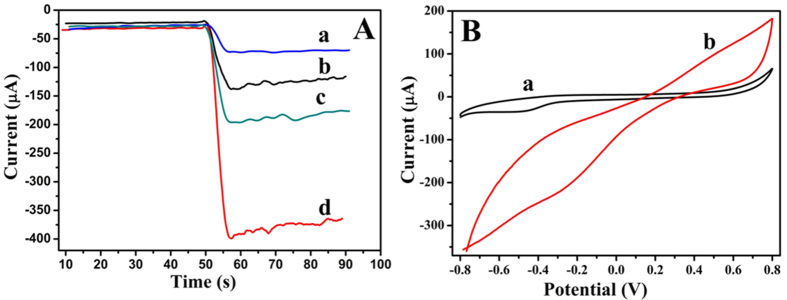
(**A**) Electrocatalytic current responses towards 5 mmol/L H_2_O_2_ of 2 mg/mL of Pt-NGs (curve a), NiPt-NGs (curve b) AuPt-NGs (curve c) and NiAuPt-NGs (curve d). (**B**) Cyclic voltammograms of bare GCE (curve a) and NiAuPt-NGs modified electrode (curve b) in PBS.

In order to further prove the electrochemical performance of the NiAuPt-NGs nanocomposites, Pt-NGs, NiPt-NGs AuPt-NGs nanocomposites were modified onto GCE respectively. The electrocatalytic responses of Pt-NGs (curve a), NiPt-NGs (curve b) AuPt-NGs (curve c) and NiAuPt-NGs (curve d) at −0.4 V in PBS at pH = 7.4 toward H_2_O_2_ were shown in Fig. 3A. The 5 μL, 5 mmol/L of H_2_O_2_ was injected into PBS at 50 s after the background current was stabilized. As expected, 2 mg/mL of NiAuPt-NGs nanocomposites show the highest current change which is much higher than 2 mg/mL of NiPt-NGs and AuPt-NGs nanocomposites. And as shown, the catalytic ability for H_2_O_2_ of the bimetallic nanocomposites is much better than the Pt-NGs nanocomposites. This also proves that NiAuPt-NGs nanocomposites have the strongest catalytic ability for H_2_O_2_. And the excellent electrochemical performance of the NiAuPt-NGs nanocomposites is beneficial to improve the sensitivity of the proposed immunosensor.

In order to further investigate the sensitivity of the prepared NiAuPt-NGs nanocomposites toward H_2_O_2_, cyclic voltammetry was used to record the electrochemical signal of the modified electrode. 6 μL, 2 mg/mL of NiAuPt-NGs solution was coated onto the electrode surface to prepare the modified electrode. After dried, the modified electrode was obtained. After the addition of the same concentration of H_2_O_2_ (5 mmol/L) in PBS at pH = 7.4, the reduction current was observed with the NiAuPt-NGs modified electrode which is much higher than the bare GCE, as shown in Fig. 3B. It proves that NiAuPt-NGs have good electrocatalytic property towards H_2_O_2_ and it also indirectly indicating that the synergetic effect is present in the NiAuPt-NGs.

### Optimization of experimental conditions

**Figure 4:**
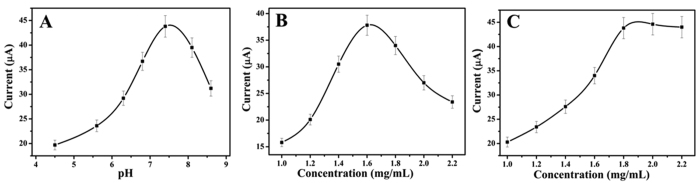
(**A**) The optimization of experimental conditions with pH. (**B**) CD-NGs concentration. (**C**) NiAuPt-NGs@Ab_2_ concentration.

The optimization of experimental conditions is necessary for obtaining the optimal electrocatalytic current response. The main effect of these experimental conditions is the value of pH, since the solution of acidic or alkaline may damage the antigen-antibody linkage and inactivate the activity of biomolecules^29^,^30^. In other words, the antigens and antibodies will keep their bioactivity in the approximate neutral conditions of pH^31^. The Fig. 4A shows the electrocatalytic current responses of the proposed immunosensor in different pH values of PBS. From this figure, the optimal electrocatalytic current response was achieved at pH = 7.4. Therefor the pH = 7.4 PBS solution was selected as the electrolyte throughout this study.

In this study, the concentration of CD-NGs has an important effect on the immunosensor, so it can’t be ignored. If there are not enough CD-NGs the quantity of captured Ab_1_ will decrease on the surface of electrode. If too much CD-NGs modified on the surface of electrode the electrical transmission from the electrode to electrolytic solution could be blocked. The Fig. 4B shows the electrocatalytic current responses of different concentration of CD-NGs in PBS at pH = 7.4. As shown in this figure, the CD-NGs at 1.6 mg/mL has the strongest electrocatalytic current responses. Therefor, 1.6 mg/mL of CD-NGs was used to conduct the electrochemical tests.

In addition, the concentration of NiAuPt-NGs@Ab_2_ is another important factor for the electrochemical tests. The Fig. 4C shows the electrocatalytic current responses of different concentration of NiAuPt-NGs@Ab_2_ for the determination of 1 ng/mL CEA in PBS at pH = 7.4. In this figure, from 1.2 to 1.8 mg/mL the value of electrocatalytic current response change rise rapidly and then reach a plateau. Using 2.2 mg/mL or 1.8 mg/mL of NiAuPt-NGs@Ab_2_ the effects of both were equivalent. Therefor, 1.8 mg/mL NiAuPt-NGs@Ab_2_ was used to conduct the electrochemical test.

### Characterization of the immunosensor

**Figure 5:**
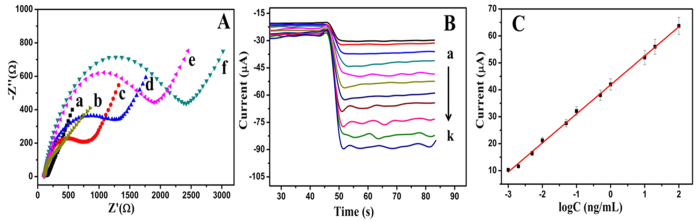
(**A**) The impedance spectra for the process of modifying the electrode, the bare GCE (a), CD-NGs/GCE (b), CD-NGs@Ab_1_/GCE (c), BSA/CD-NGs@Ab_1_/GCE (d), CEA/BSA/CD-NGs@Ab_1_/GCE (e) and NiAuPt-NGs@Ab_2_/CEA/BSA/CD-NGs @Ab_1_/GCE(f). (**B**) Amperometric curve of the immunosensor for the detection of different concentrations of CEA at −0.4 V (5 mmol/L H_2_O_2_). (**C**) Calibration curve of the immunosensor toward different concentrations of CEA. Error bar = RSD (n = 5).

The fabrication process of the sandwich-type electrochmical immunosensor can be monitored by electrochemical impedance spectroscopy. It is mainly to characterize the interface properties of the modified electrodes. Figure 5A shows the impedance spectra for the process of modifying the electrode, which were recorded from 1 to 10^5 ^Hz at 0.2 V in a solution containing 0.1 mol/L KCl and 2.5 mol/L Fe(CN)_6_^3−^/Fe(CN)_6_^4−^. The impedance spectra include two parts, the linear portion and the semicircular portion. The linear portion at low frequencies is associated with electrochemical behavior limited by diffusion. And the semicircle portion at high frequencies is associated with the electrochemical process subject to electron transfer which the diameter corresponds to the resistance. In short, the change of the resistance can be judged by the diameter of semicircle portion. As shown in Fig. 5A, the bare GCE presents extremely small resistance (curve a). After CD-NGs nanocomposite modified on the electrode (curve b), the resistance has not obviously increased compared with the bare GCE. After incubation with Ab_1_ (curve c), the resistance was significantly increased, indicating that Ab_1_ was well immobilized on the electrode successfully. In the same way, when the BSA modified on the electrode (curve d), the resistance was significantly increased. The possible reason is that the protein molecules could block the transfer of electrons. Similarly, the resistance further increased with the CEA modified on the electrons (curve e). When NiAuPt-NGs@Ab_2_ (curve f) was immobilized on the surface of CEA/BSA/Ab_1_@CD-NGs/GCE the resistance increased to the maximum which indicates the immunosensor was successfully modified layer by layer on the electrode. Based on this result, we conclude that the electron transfer ability of the modified electrodes is determined by the conductive nanocomposite and the non-conductive bioactive substances.

Under the optimal conditions, the proposed sandwich-type electrochemical immunosensor which using CD-NGs as signal amplification platform and NiAuPt-NGs nanocomposites as labels was used to detect different concentrations of CEA by amperometric i-t curve in PBS the pH = 7.4 at −0.4 V. The Fig. 5B shows the electrocatalytic current responses of the proposed immunosensor for the determination of CEA. From (a) to (k) these curves respectively represent the electrocatalytic current responses of the immunosensor for the determination of CEA: 0.001, 0.002, 0.005, 0.01, 0.05, 0.1, 0.5, 1, 10, 20, 100 ng/mL. The Fig 5C shows the relationship between electrocatalytic current responses of the proposed immunosensor and the concentration of CEA within the range of 0.001 to 100 ng/mL. The linear regression equation of the calibration curve is I = 41.85 + 10.87 logC with correlation coefficient of 0.9982 and the proposed immunosensor expressed an extremely low limit of detection of 0.27 pg/mL. The positive results might be attributed to NiAuPt-NGs nanocomposites which could conjugated to amounts of capture antibodies and the synergetic effect present in NiAuPt-NGs that favors electron transfer. They could greatly increase the response to H_2_O_2_, broaden the scope of testing and lead to higher sensitivity.

In addition, the fabricated immunosensor based on the trimetallic NiAuPt-NGs nanocomposites was cmpared with other immunosensors by different methods and with other electrochemical immunosensor which used other nanomaterials as the labels (Supplementary Tables S1 and S2). The proposed immunosensor shows a wide linear range and an extremely low detection limit.

### Reproducibility selectivity and stability of the immunosensor

A series of immunosensors which were fabricated on five different electrodes were prepared for the determination of 1 ng/mL of CEA to evaluate the reproducibility of the immunosensor. The relative standard deviation (RSD) of the five measurements at 1 ng/mL CEA was calculated to be 3.2%, proving the acceptable reproducibility of the detection signal.

**Figure 6:**
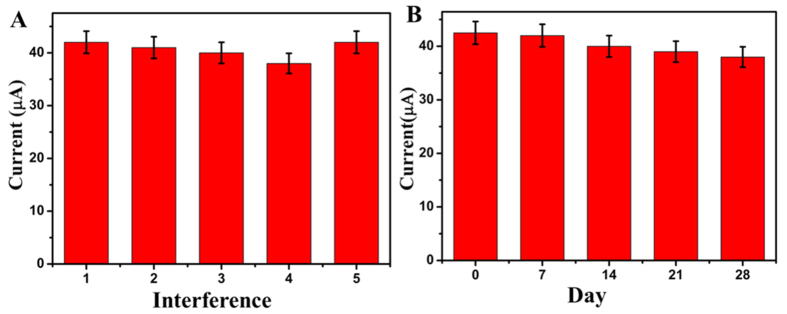
(**A**) Amperometric response of the immunosensor to 1.0 ng/mL CEA + 30 ng/mL BSA (1), 1.0 ng/mL CEA + 30 ng/mL glucose (2), 1.0 ng/mL CEA + 30 ng/mL SCCA (3), 1.0 ng/mL CEA + 30 ng/mL AFP (4), 1.0 ng/mL CEA + 30 ng/mL vitamin C (5). (**B**) The stability study of the CEA immunosensor. (Error bar = RSD, n = 5).

We used BSA, glucose, squmaous cell carcinoma antigen (SCCA), alpha fetal protein (AFP), vitamin C as interference substances to examine the selectivity of the immunosensor. 30 ng/mL of interference solution with 1 ng/mL of CEA was measured by the proposed immunosensor and the result was depicted in Fig. 6A. The variation of the electrocatalytic current response was less than 5% of that without the interference, proving the selectivity of the proposed immunosensor was acceptable.

The stability of the immunosensor is an important part for the using of immunosensor in clinical detection. A series of prepared immunosensors were stored at 4 °C, and the result was depicted in Fig. 6B. After 7 days, the current response of the immunosensor had little change, and after 28 days, the current response retained 89.4%. Thus, the immunosensor has acceptable storage stability. According to the results of the above, the reproducibility, selectivity and stability of the proposed immunosensor are all acceptable. So the immunosensor could be used to detect CEA in real human sample.

### Real sample analysis

Based on the above research, the proposed immunosensor could be used to detect the content of CEA in the serum. In order to further prove the reliability of the immunsensor, the recovery of different concentrations of CEA in serum sample was detected by standard addition method. And the serum sample was obtained by centrifugation from human blood, before used it was stored at −16 °C. The results are shown in Table 1. The RSD is in the range 3.97% to 5.30% and the recovery is in the range 99.5% to 1.04%, and the good accuracy can be obtained of the proposed method for sample detection.

## Conclusions

This work has proposed an ultrasensitive sandwich-type electrochemical immunosensor based on a novel signal amplification strategy by employing CD-NGs as the matrix and NiAuPt-NGs as the labels for the quantitative determination of CEA. The immunosensor displayed an extremely low limit of detection, a wide detection range, good selectivity, acceptable reproducibility and stability. This is in large part due to the CD-NGs which has large specific surface area to capture a large amount of anti-CEA. And NiAuPt-NGs have large specific surface area, good biocompatibility, high conductivity and excellent electrochemical catalysis towards H_2_O_2_, which greatly increase the probability of antibody-antigen interactions and beneficial for improving sensitivity of the immunosensor. The proposed immunosensour showed excellent analytical performance for the measurement of CEA providing a promising application for CEA in clinical diagnostics.

## Materials and Methods

### Reagents and apparatus

Human CEA, the anti-CEA and secondary anti-CEA were purchased from Beijing Dingguochangsheng Biotechnology Co. Ltd. (China). Bovine serum albumin (BSA, 96–99%) was purchased from Sigma (USA). Phosphate buffered saline (PBS) which was prepared by 1/15 mol/L Na_2_HPO_4_ and KH_2_PO_4_ with various volume ratio was used as an electrolyte for all electrochemical measurements. Ultrapure water was used throughout the experiments and all other chemicals were of analytical reagent. All electrochemical measurements were carried out using a CHI 760D electrochemical workstation (Shanghai Chenhua Instrument Co., Ltd., China). Scanning electron microscopy (SEM) images and energy dispersive X-ray spectral data (EDX) were obtained using a Quanta FEG250 field emission environmental SEM. Fourier transform infrared spectroscopy (FTIR) spectrum was obtained from VERTEX 70 (Germany).

### Preparation of CD-NGs

In this group, GO was synthesized by a modified Hummers’ method^32^. The CD-NGs was synthesized by the following method. 40 mL of aqueous solution containing 10 mg of GO and 80 mg of β-CD was mixed with 300 μL of ammonia solution. Then 20 μL of hydrazine hydrate (80 wt%) was added under stirring at 60 °C for 4 h. The CD-NGs was obtained by centrifugation (10000 rpm/min) for 10 min then dried under vacuum.

### Preparation of NiAuPt-NGs

The metal nanoparticles on NGs were prepared by the simultaneous reduction of GO and metal precursors with NaBH_4_. The concentration of each precursor, NiCl_2_, H_2_PtCl_6_, and HAuCl_4_, was 0.002 mol/L, and the GO concentration was 0.5 mg mL^−1^ in water (50 mL). The mixture was ultrasonicated for 30 min, followed by stirring for 1 h. The pH value of the solution was adjusted to 8–9 by adding 1 mol/L KOH aqueous solution. 30 mL of 0.05 mol/L NaBH_4_ was added dropwise, then the solution was refluxed in a water bath at 75 °C for 4 h. The yellowish brown solution turned black. The black NiAuPt-NGs were rinsed with copious ultrapure water and finally dried under vacuum at 35 °C for 12 h. We also synthesized NGs, Pt-NGs, AuPt-NGs, and NiPt-NGs through a similar procedure. The molar ratio of the metal precurors for binary metal nanoparticles was also kept at 1:1.

### Preparation of NiAuPt-NGs@Ab_2_ labels

The mixture solution of NiAuPt-NGs (1.8 mg/mL, 1 mL), Ab_2_ (10 μg/mL, 1 mL) was well mixed and stirred for 24 h at 4 °C. After centrifugation, the resulting NiAuPt-NGs@Ab_2_ labels were dispersed in 1 mL of PBS at pH = 7.4 and stored at 4 °C.

### Fabrication of the immunosensor

A glassy carbon electrode (GCE) was polished to a mirror-like finish with 0.3 and 0.05 μm alumina powder then thoroughly cleaned before use by ultrapure water. First, an aqueous solution of CD-NGs (1.6 mg/mL, 6 μL) was added onto the surface of GCE then dried. Ab_1_ dispersion (10 μg/mL, 6 μL) was added onto the CD-NGs/GCE stored at 4 °C for 1 h then washed carefully. After incubated, BSA solution (10 mg/mL, 3 μL) was added onto the Ab_1_/CD-NGs/GCE to eliminate nonspecific binding sites. After incubated for another 1 h at 4 °C, the BSA/Ab_1_/CD-NGs/GCE was washed and incubated with a varying concentration of CEA (0.001–100 ng/mL, 6 μL) for 1 h at 4 °C, and then the CEA/BSA/Ab_1_/CD-NGs/GCE was washed extensively to remove unbounded CEA molecules. Finally, the prepared NiAuPt-NGs@Ab_2_ labels solution (1.8 mg/mL, 6 μL) was added onto the modified electrode surface for 1 h at 4 °C, and the NiAuPt-NGs@ Ab_2_/CEA/BSA/Ab_1_/CD-NGs/GCE was washed thoroughly before measurement.

### Detection of CEA

A conventional three-electrode system was used for all electrochemical measurements: the GCE with a diameter of 4 mm as the working electrode, a saturated calomel electrode (SCE) as the reference electrode, and a platinum wire electrode as the counter electrode. For amperometric i-t curve to record the amperometric response, and a detection potential of −0.4 V was selected. The PBS at 7.4 was used as electrolyte for all electrochemical measurements. The 5 μL, 5 mmol/L of H_2_O_2_ was added into the PBS after the back ground current was stabilized.

## Additional Information

**How to cite this article**: Tian, L. *et al*. Ultrasensitive sandwich-type electrochemical immunosensor based on trimetallic nanocomposite signal amplification strategy for the ultrasensitive detection of CEA. *Sci. Rep.* **6**, 30849; doi: 10.1038/srep30849 (2016).

## Supplementary Material

Supplementary Information

## Figures and Tables

**Figure 1:**
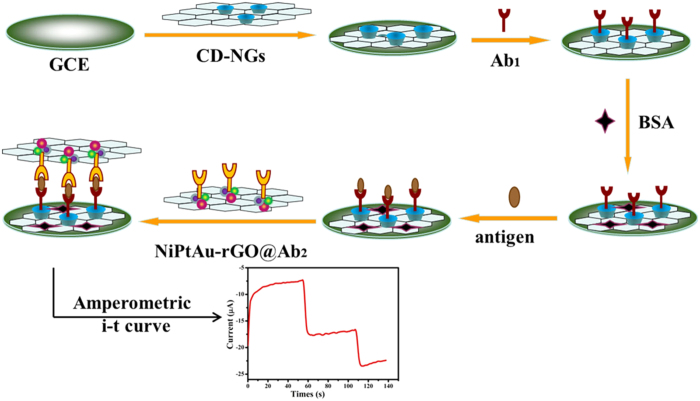
The schematic illustration of the fabrication process of the sandwich-type electrochemical immunosensor.

**Table 1:** Determination of CEA in human serum samples.

**Initial CEA concentration in sample** (**ng/mL**)	**Added CEA concentration** (**ng/mL**)	**Average value** (**ng/mL**)	**RSD** (**%**,**n** = **5**)	**Recovery** (**%**,**n** = **5**)
	1.00	1.53	5.30	1.04
0.49	2.00	2.48	4.02	0.995
	3.00	3.48	3.97	0.997
