# Supplementary material for: Ultrasensitive sandwich-type electrochemical immunosensor based on trimetallic nanocomposite signal amplification strategy for the ultrasensitive detection of CEA
Source: Sci Rep. 2016 Aug 4;6:30849. doi: 10.1038/srep30849 (PMC4973229; doi:10.1038/srep30849)
Supplement: Supplementary Information [file srep30849-s1.pdf]

Supplementary Information

Ultrasensitive sandwich-type electrochemical immunosensor based on trimetallic nanocomposite signal amplification strategy for the ultrasensitive detection of CEA

Lihui Tian, Li Liu, Yueyuan Li, Qin Wei, Wei Cao*

Key Laboratory of Chemical Sensing & Analysis in Universities of Shandong, School of Chemistry and Chemical Engineering, University of Jinan, Jinan 250022, PR China

Lihui Tian (E-mail: jndx_Tianlihui@163.com)

Li Liu (E-mail: liul_jndx@163.com)

Yueyuan Li (E-mail: yueyuanli86@163.com)

Qin Wei (E-mail: sdjndxwq@163.com)

Wei Cao (E-mail: jn_chm302@163.com)

*Corresponding author. Tel.: +86-531-82767890. Fax: +86-531-82765475.

E-mail address: jn_chm302@163.com (jncw88@163.com)

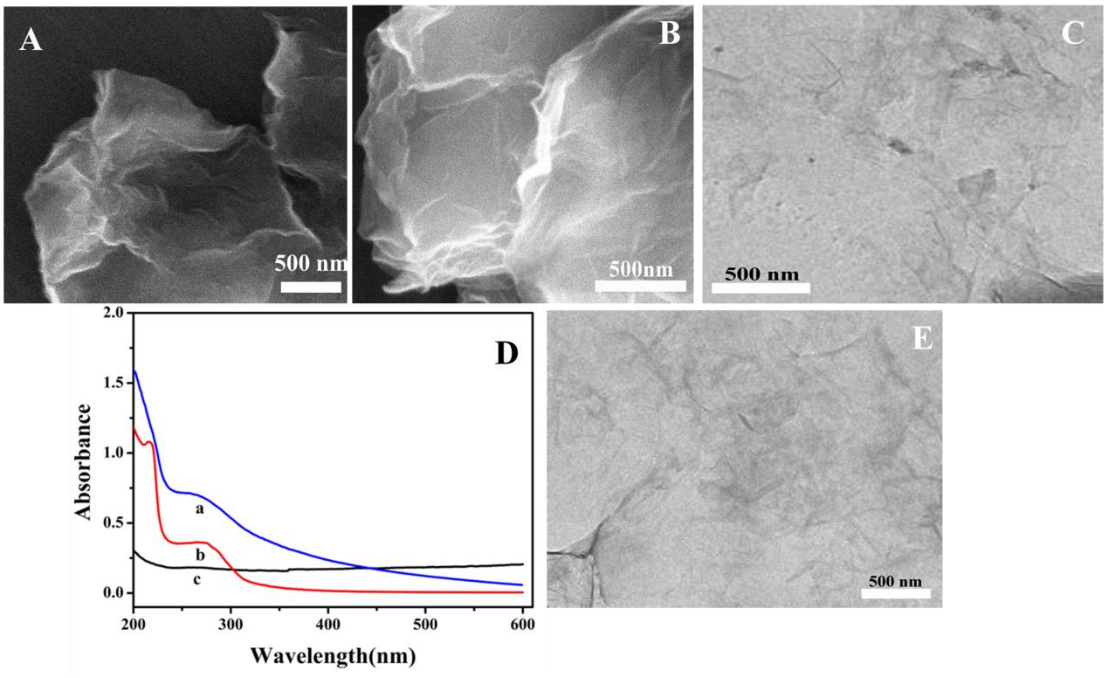

Figure S1 SEM images of NGs (A) and CD-NGs (B); The TEM of NGs (C) and CD-NGs (E); (D) The UV-vis spectra of CD-NGs (a), β-CD (b) and NGs (c);

Table S1 Comparison of the performance of the proposed electrochemical CEA immunosensor for other reports

Nanomaterials	Marker	Linear range (ng/mL)	Limit of Detection (pg/mL)	Reference
PdPt nanocages/MWCNT-Ab_2	CEA	0.001-20	0.2	1
Ab_2-Au-TB-rGO	CEA	0.01-100	3	2
Ag-Ab_2	CEA	0.001-50	0.27	3
Fe_3O_4/Au-Ab_2	CEA	0.001-30	0.39	4
HRP-Ab_2-PtNPs	CEA	0.02-120	12	5
Ab_2-NiAuPt-NGs	CEA	0.001-100	0.27	This work

Table S2. Comparison of different methods for the detection of CEA

Methods	Linear range	Limit of detection	References
FL quenching method	0.257~12.9 ng/mL	5 pg/mL	6
Paper-based microfluidic electrochemical	0.01~100 ng/mL	0.01 ng/mL	7
Electrochemical immunosensor	0.001~20 ng/mL	0.2 pg/mL	1
ECL immunosensor	20 fg/mL~1.0 ng/mL	6.7 fg/mL	8
Electrochemical immunosensor	0.001~100ng/mL	0.27 pg/mL	This method

References

1. Li, N. *et al*. An ultrasensitive electrochemical immunosensor for CEA using MWCNT-NH_2 supported PdPt nanocages as labels for signal amplification. *J. Mater. Chem. B.* **3,** 2006-2011 (2015).
2. Liu, N., Liu, Z., Han, H. & Ma, Z. Graphene oxide reduced directly by redox probes for multiplexed detection of tumor markers. *J. Mater. Chem. B.* **2**, 3292-3298 (2014).
3. Wang, X. *et al*. An ultrasensitive electrochemical immunosensor based on the catalytical activity of MoS2-Au composite using Ag nanospheres as labels. *Sensor. Actuat. B-Chem.* **206,** 30-36 (2015).
4. Dong, P., Liang, R., Huang, H. & Qiu, J. Electrochemical immunosensor for carcinoembryonic antigen based on signal amplification strategy of graphene and Fe_3O_4/Au NPs. *J. Electroanal. Chem.* **761,** 112–117 (2016).
5. Yang. H. *et al*. Electrochemical immunosensor for detecting carcinoembryonic antigen using hollow Pt nanospheres-labeled multiple enzyme-linked antibodies as labels for signal amplification. *Biochem. Eng. J.* **56,** 116–124 (2011).
6. Zhou, Z. M. *et al*. Carcino-embryonic antigen detection based on fluorescence resonance energy transfer between quantum dots and graphene oxide. *Biosens. Bioelectron.* **59,** 397-403 (2014).
7. Wu, Y., Xue, P., Hui, K. M. & Kang, Y. A paper-based microfluidic electrochemical immunodevice integrated with amplification-by-polymerization for the ultrasensitive multiplexed detection of cancer biomarkers. *Biosens. Bioelectron*. **52,** 180-187 (2014).
8. Zhuo, Y. *et al*. Sandwich-format electrochemiluminescence assays for tumor marker based on PAMAM dendrimer-L-cysteine-hollow gold nanosphere nanocomposites. *Biosens. Bioelectron.* **53,** 459-464 (2014).
